# Case Report: Disseminated *Mycobacterium abscessus* subsp*. bolletii* infection with central nervous system involvement in acquired anti-IFN-γ autoantibody syndrome

**DOI:** 10.3389/fimmu.2026.1726291

**Published:** 2026-03-11

**Authors:** Lina Li, Lijun Zhang, Chaowen Deng, Haibing Xiao

**Affiliations:** 1 Department of Neurology, Neuromedicine Center, The University of Hong Kong-Shenzhen Hospital, Shenzhen, Guangdong, China; 2 Shenzhen Clinical Research Center for Rare Diseases, Shenzhen, China; 3 Department of Rheumatology, The University of Hong Kong-Shenzhen Hospital, Shenzhen, China; 4 Department of Infectious Diseases and Microbiology, The University of Hong Kong-Shenzhen Hospital, Shenzhen, China

**Keywords:** anti-interferon-gamma autoantibody, central nervous system infection, cyclophosphamide, *Mycobacterium abscessus* subsp. *bolletii*, Sweet’s syndrome

## Abstract

Acquired Anti-interferon (IFN)-γ Autoantibody Syndrome (AAS) is an emerging immunodeficiency predisposing to disseminated nontuberculous mycobacterial infections. We report a 51-year-old woman with AAS presenting with Sweet’s syndrome, disseminated *Mycobacterium abscessus* subsp*. bolletii* infection, and central nervous system (CNS) involvement manifested as leptomeningeal enhancement despite sterile cerebrospinal fluid. Diagnosis was confirmed by serum anti-IFN-γ autoantibody titer >1:10,000 and lymph node culture. The patient achieved sustained remission through a staged approach: 26 months of tailored antibiotics (imipenem/ceftazidime/amikacin/clarithromycin-based regimen) followed by delayed cyclophosphamide immunotherapy. This case highlights the importance of anti-IFN-γ autoantibody screening in disseminated infections and demonstrates that CNS involvement may occur without typical cerebrospinal fluid abnormalities. Our management strategy-prioritizing infection control before immunosuppression-provides a pragmatic framework for resource-limited settings.

## 1 Introduction

Disseminated *Mycobacterium abscessus* infections primarily occur in immunocompromised hosts (1). Recent studies identify acquired anti-interferon (IFN)-γ autoantibody syndrome (AAS) associated immunodeficiency, an acquired immunodeficiency mediated by neutralizing autoantibodies, as an increasingly recognized etiology for disseminated mycobacterial infections, salmonellosis and herpes zoster reactivation, frequently associated with Sweet’s syndrome (2–4). This association reflects antibody-mediated neutralization of IFN-γ, leading to impaired Th1 immunity and aberrant neutrophil recruitment in Sweet’s syndrome (5). Herein, we report a case of disseminated *M. abscessus* subsp*. bolletii* infection with AAS to discuss the diagnostic and management insights, particularly regarding central nervous system (CNS) involvement and the use of cyclophosphamide (CTX).

## 2 Case report

A 51-year-old woman presented with recurrent fever and skin rash for one month, accompanied by headache for 3 days. Upon referral to our department, the patient had been treated elsewhere for unknown fever, dry cough, and generalized maculopapular rash. Initial investigations included complete blood picture showing significant leukocytosis (predominantly neutrophilia) and chest computed tomography (CT) revealing bilateral pulmonary infiltrates with mediastinal lymphadenopathy. After two weeks of empirical antibiotics therapy with moxifloxacin and linezolid, which failed to resolve her condition, symptoms recurred and progressed to headache and joint pain. Physical examination found body temperature was 38 degrees Celsius, blood pressure was 122/85 mmHg, heart rate was 89 beats per minute. Popular eruptions were noted on the face and extremities. The superficial lymph nodes throughout the body were found to be enlarged, including those in the neck, the subclavian region, and the groin. The stiff-neck indicated meningeal inflammation.

Investigations: Complete blood picture: significant leukocytosis (White blood cell count 28.5×10^9^/L, normal range 3.5-9.5×10^9^/L), elevated C-reactive protein 66.79 mg/L (normal range <5 mg/L) and erythrocyte sedimentation rate 117 mm/h (normal range <20 mm/h) indicate a state of intense inflammatory response. HIV serology, syphilis serology, tumor markers, and lymphocyte subsets were unremarkable. ANA was positive (1:320) while ENA profile was negative.

Microbiology: Four sets of blood cultures were negative after 14 days incubation. For persistent headache, fever, and stiff-neck, lumbar puncture was performed to evaluate for intracranial infection. Cerebrospinal fluid (CSF) analysis shown opening pressure was 150 mm H_2_O (normal range 80–180 mm H_2_O). Nucleus cell count: 9 cells/μL (normal range 0–5 cells/μL). Biochemistry normal (Glucose 3.3 mmol/L (simultaneous blood glucose 4.9 mmol/L), protein 0.3 g/L). CSF polymerase chain reaction was negative for *Mycobacterium tuberculosis*, herpes simplex virus, varicella-zoster virus, cytomegalovirus, and enterovirus. Extended 4-week CSF culture and metagenomic next-generation sequencing were also negative.

**Figure 1:**
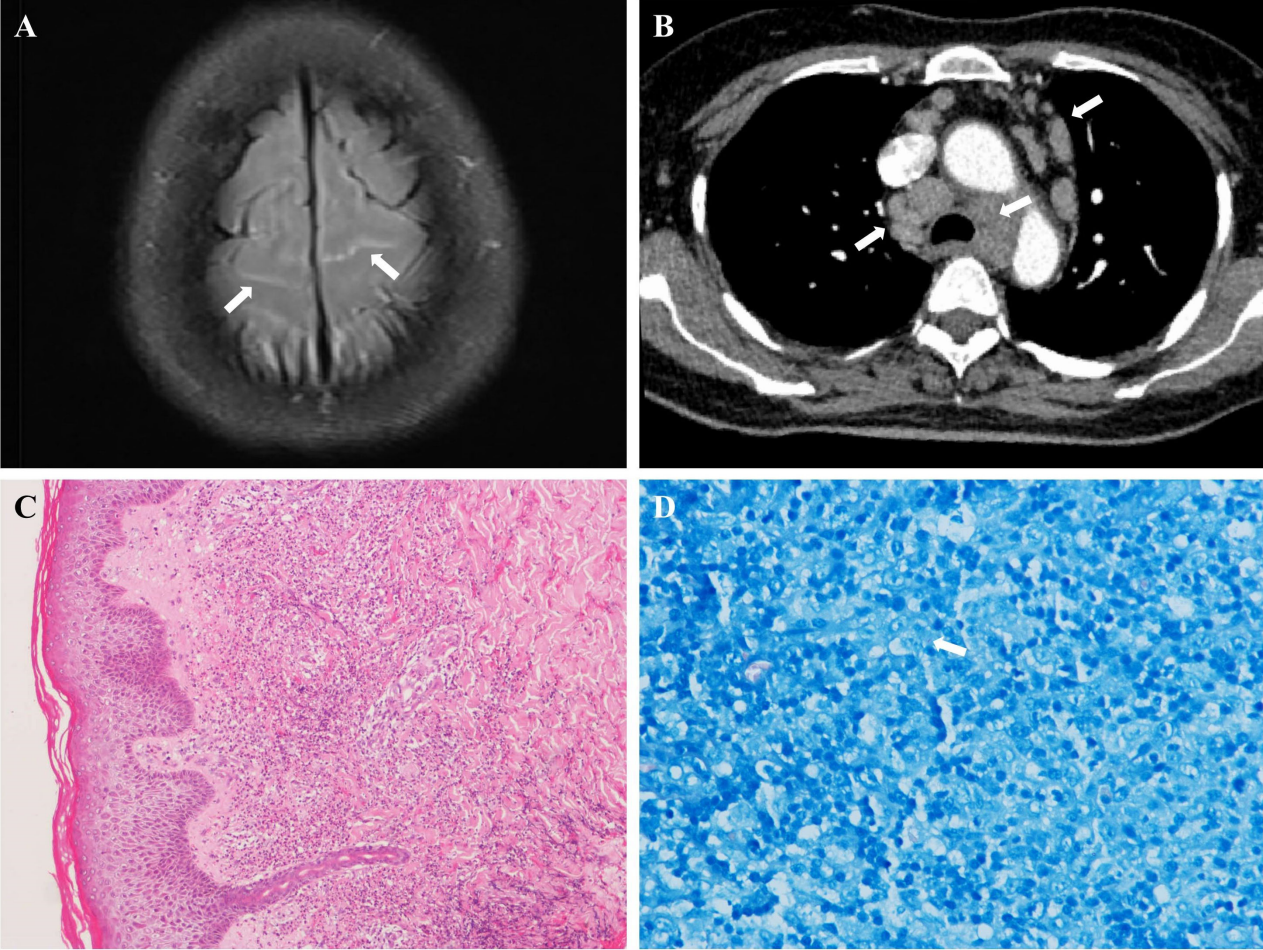
Diagnostic findings in acquired anti-interferon-γ autoantibody syndrome with disseminated *Mycobacterium abscessus* infection. **(A)** Brain MRI (post-contrast T2-FLAIR fat-suppressed sequence) showing linear leptomeningeal enhancement (arrows). **(B)** Thoracoabdominal CT demonstrating extensive lymphadenopathy (arrows). **(C)** Skin biopsy (H&E, 100×) with neutrophilic dermal infiltrate, consistent with Sweet’s syndrome. **(D)** Acid-fast bacilli (arrows) in lymph node tissue (Ziehl-Neelsen stain, 40×).

Imaging: Brain magnetic resonance imaging (MRI) contrast showed linear leptomeningeal enhancement along the superficial aspects of bilateral frontoparietal gyri (**Figure 1A**, arrows), consistent with localized leptomeningitis. No parenchymal abnormalities or pachymeningeal involvement were observed. Thoracoabdominal CT revealed extensive lymphadenopathy (**Figure 1B**, arrows).

Histopathology: Skin biopsy (right foot) showed dense dermal neutrophilic infiltrate without vasculitis, Sweet’s syndrome was considered (**Figure 1C**). Cervical lymph node biopsy revealed necrotizing granulomas with neutrophilic abscesses, and Acid-fast bacilli was found on Ziehl-Neelsen stain (**Figure 1D**). Subsequently, *M. abscessus* was isolated on mycobacterial culture after 5 days of incubation. Identification via matrix-assisted laser desorption/ionization time-of-flight mass spectrometry (MALDI-TOF MS) confirmed the subspecies as *M. abscessus* subsp. *bolletii.*

Serology: Serum anti-IFN-γ autoantibodies were detected using a two-step approach as previously described (6) with minor modifications. 1. Screening enzyme-linked immunosorbent assay (ELISA). Recombinant human IFN-γ (R&D Systems) was coated onto Nunc immunoplates (Nalge Nunc International). After blocking with 5% normal goat serum, patient serum diluted 1:1000 was added and incubated. Bound antibodies were detected with horseradish peroxidase-conjugated goat anti-human total Ig (Zymed) or anti-human IgG (Biosource). Optical density was measured at 450 nm. This step qualitatively confirmed the presence of anti-IFN-γ autoantibodies. 2. Plasma-spiking assay for IFN-γ-binding capacity (titer determination). To estimate neutralizing capacity, a plasma-spiking assay was performed. This method measures residual free IFN-γ after incubation with serial serum dilutions and has been widely accepted as a surrogate for neutralization in anti-IFN-γ autoantibody syndrome (3, 4, 6). Serial dilutions of the patient’s serum (1:100, 1:200, 1:1000, 1:10 000, 1:20 000, and 1:40 000) were incubated with a fixed amount of IFN-γ (1000 pg). Residual free IFN-γ was quantified using a commercial ELISA kit (BD OptEIA). The titer was defined as the highest serum dilution that reduced free IFN-γ by ≥50% (IC_50_). All assays were run in duplicate with appropriate controls. Positive controls consisted of three known high-titer sera. For routine experiments, a single healthy donor serum diluted 1:1000 was included as the negative control; the test was established during assay validation using sera from 1000 healthy donors. Disease controls comprised four NTM-infected patients without immunodeficiency. The screening ELISA was positive at 1:1000, and the plasma-spiking assay determined the titer to be >1:10,000.

**Figure 2:**
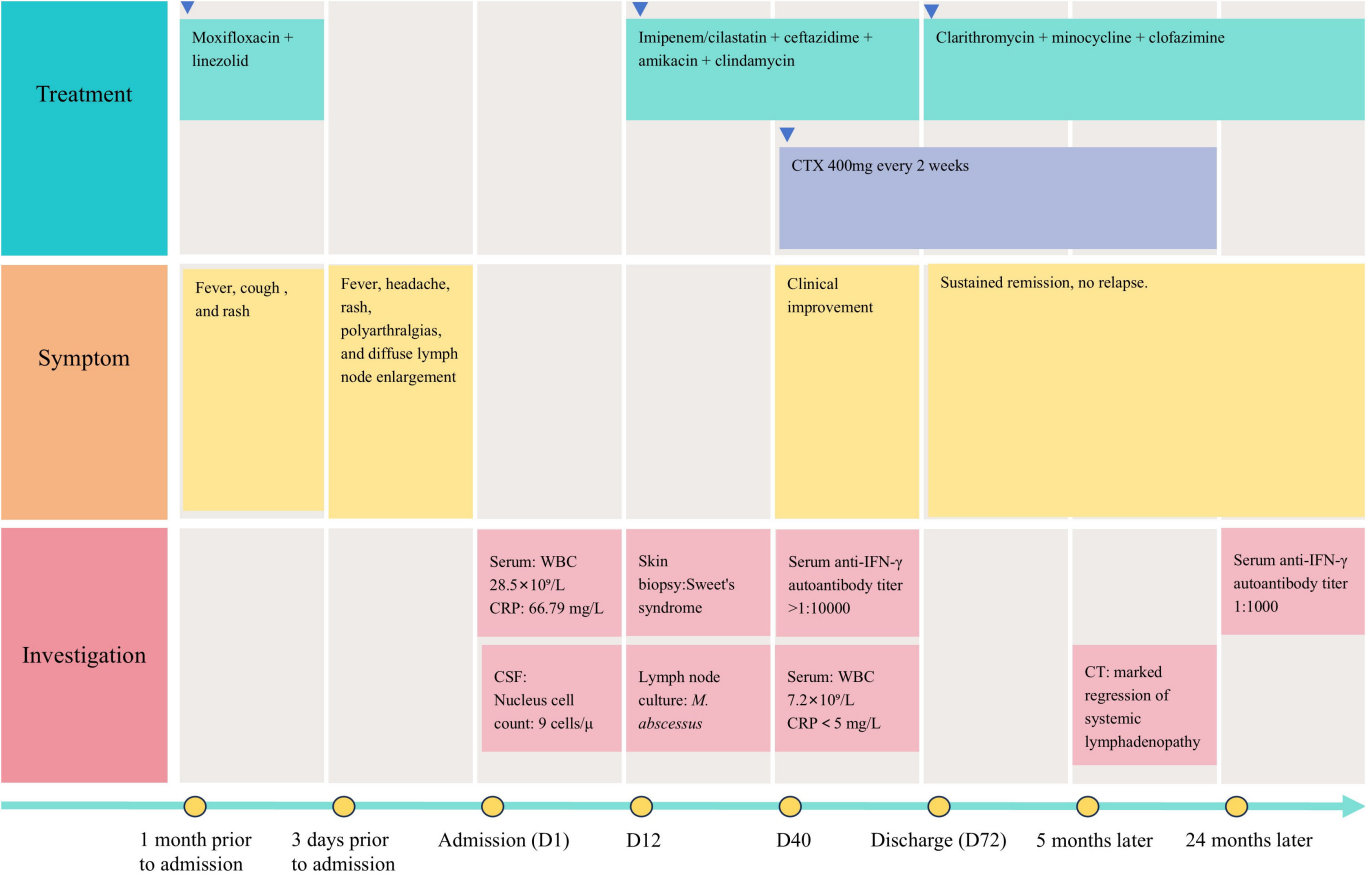
Timeline of the patient’s clinical trajectory and therapeutic Interventions. WBC, white blood cell count; CRP, C-reactive protein; CSF, cerebrospinal fluid; D, day; CTX, cyclophosphamide; CT, computed tomography.

Treatment and clinical outcomes (**Figure 2**): Based on the sensitivity test results (**Table 1**) of the isolated *M. abscessus*, the antibiotics regimens are as follows: Intensive Phase (2 months): The regimen included imipenem/cilastatin (500 mg every 6 hours), ceftazidime (1 g every 8 hours), amikacin (15 mg/kg per day), and clindamycin (500 mg every 12 hours).

**Table 1:** Results of antimicrobial susceptibility testing for *Mycobacterium abscessus* subsp*. bolletii* strain isolated from the lymph node.

Antibiotics agent	Diameter of inhibition zone (mm)	Susceptibility Categories
Ceftazidime	6	R
Amikacin	22	S
Tobramycin	6	R
Ciprofloxacin	6	R
lmipenem	16	I
Tigecycline	36	S
Levofloxacin	6	R
Clarithromycin	46	S
Doxycycline	6	R
Minocycline	28	S
Linezolid	6	R
Chloramphenicol	6	R
lmipenem plus ceftazidime	23	

S, Susceptible; R, Resistant. Susceptibility testing was performed using Kirby-Bauer Disk Diffusion method.

Consolidation Phase (24 months): The regimen was adjusted to clarithromycin (500 mg twice daily), minocycline (100 mg twice daily), and clofazimine (100 mg once daily).

Intravenous CTX (400 mg every 2 weeks for 6 months) was initiated 4 weeks after antimicrobial therapy onset, once C-reactive protein normalized (<5 mg/L) and white blood cell count declined to 7.2×10^9^/L (normal range 3.5-9.5×10^9^/L).

Serum anti-IFN-γ autoantibody titer declined to 1:1000 at 24 months. Follow-up at 5, 12, and 24 months showed sustained clinical remission and normalized inflammatory markers. Repeat imaging after six months of antimicrobial therapy demonstrated marked regression of systemic lymphadenopathy.

## 3 Discussion

AAS is an acquired immunodeficiency disorder characterized by high-titer neutralizing antibodies against IFN-γ, a pivotal cytokine for macrophage activation and intracellular pathogen clearance (3). This immune dysfunction predisposes patients to disseminated opportunistic infections such as *M. abscessus (*2). Our patient presented with the classic diagnostic triad: (1) biopsy-confirmed Sweet’s syndrome, (2) culture-proven disseminated *M. abscessus* infection, and (3) significantly elevated serum anti-IFN-γ autoantibody titer (>1:10,000). The immunopathogenesis involves antibody-mediated blockade of IFN-γ receptor binding, which disrupts JAK-STAT1 signaling and subsequent IL-12 production (3, 7). This molecular interference leads to impaired M1 macrophage polarization, resulting in deficient generation of reactive oxygen species (ROS) and nitric oxide (NO) - critical mediators for intracellular microbial killing (8).

While CNS involvement remains uncommon, our case demonstrates three distinctive features: (1) sterile CSF despite radiographic evidence of leptomeningeal enhancement, (2) marked dissociation between profound peripheral leukocytosis and minimal CSF pleocytosis, and (3) focal rather than diffuse leptomeningeal involvement on MRI (**Figure 2A**). These observations may reflect early-stage neuroinvasion prior to complete blood-brain barrier disruption, as IFN-γ blockade downregulates RhoA/Rho-associated coiled-coil-containing protein kinase (ROCK) signaling, reducing occludin phosphorylation and disrupting blood-brain barrier integrity (9).

Current evidence supports two strategies for antibody clearance: rituximab (anti-CD20) and CTX (10, 11). The study by Laisuan et al. demonstrated comparable efficacy between CTX and rituximab (remission rates 92% versus 88%), but CTX achieved faster seroconversion (median 84 vs 99 days) (12). Our choice of CTX was further justified by: (1) cost-effectiveness in resource-limited settings (CTX costs <10% of rituximab regimens), (2) rapid neutrophil count normalization prior to initiation. Emerging alternatives like daratumumab (anti-CD38), bortezomib show promise in refractory cases but require further study (13, 14). Critically, we initiated CTX only after achieving clear clinical and laboratory evidence of infection control, approximately 4 weeks after starting anti-NTM therapy. This cautious approach aimed to avoid the potentially catastrophic consequences of premature immunosuppression in the setting of active disseminated infection, particularly with CNS involvement. This pragmatic approach reflects real-world challenges in resource-limited settings, where comprehensive genetic profiling is often inaccessible.

This case highlights three key clinical insights for managing patient with AAS: First, routine screening for anti-IFN-γ autoantibodies should be performed in patients with disseminated mycobacterial infections, and brain MRI evaluation remains necessary even when CSF findings are unremarkable. Second, treatment should follow a staged approach initial intensive antimicrobial therapy for at least 4 weeks, followed by combined CTX immunotherapy only after infection control is achieved, with maintenance antibiotics continued for at least 12 months. Finally, in resource-limited settings, pragmatic strategies can be adopted including prioritizing cost-effective CTX over rituximab. These measures provide a feasible management framework for similar cases, particularly in underserved regions.

While this case provides important insights, several limitations merit consideration. Firstly, due to cost constraints, it was not possible to assess the HLA-DRB1*15:02 and *16:02 alleles, which are closely related to the etiology of AAS in the Asian population (15). This constitutes a limitation in the genetic risk stratification. Secondly, longitudinal monitoring of autoantibody titers and neutralizing capacity at predefined intervals (e.g., 3, 6, and 12 months) was not performed due to the patient’s financial constraints. Although a significant decline in titer was observed at 24 months, the lack of intermediate time-point data precludes a detailed analysis of the kinetics of antibody clearance in relation to clinical response. Future studies should incorporate systematic serological follow-up to better correlate antibody dynamics with treatment outcomes. Thirdly, patients with this disease require lifelong follow-up, as a two-year period is insufficient to monitor for late-stage opportunistic infections. Nevertheless, our findings establish key principles for managing such cases in resource-limited settings.

## 4 Conclusions

This case highlights AAS as a critical differential diagnosis for disseminated NTM infections with CNS involvement. The successful management requires a dual approach: prolonged antimycobacterial therapy combined with immunosuppressive treatment, with the critical caveat that immunomodulators should only be initiated after confirmed infection control. Future studies should explore CSF cytokine profiling and long-term neurocognitive outcomes in AAS-related CNS involvement.

## Data Availability

The original contributions presented in the study are included in the article/**Supplementary Material**. Further inquiries can be directed to the corresponding authors.
