# Supplementary material for: Case Report: Disseminated Mycobacterium abscessus subsp. bolletii infection with central nervous system involvement in acquired anti-IFN-γ autoantibody syndrome
Source: Front Immunol. 2026 Mar 11;17:1726291. doi: 10.3389/fimmu.2026.1726291 (PMC13013496; doi:10.3389/fimmu.2026.1726291)
Supplement: Supplementary file 1 [file Table1.docx]

**Supplementary Methods**

**Detailed Protocol for Anti-IFN-γ Autoantibody Detection**

The following protocol was adapted from Tang et al. (Clin Vaccine Immunol 2010;17:1132‑8) with minor modifications for local laboratory conditions.

**1. ELISA for anti-IFN-γ autoantibody screening**

**Reagents:**

- Recombinant human IFN-γ (R&D Systems, catalog no. 285-IF-100)

- Nunc MaxiSorp immunoplates (Thermo Fisher Scientific)

- Blocking buffer: 5% normal goat serum in PBS

- Dilution buffer: PBS containing 0.05% Tween-20 and 1% BSA

- Detection antibodies: HRP-conjugated goat anti‑human total Ig (Zymed, 62-7420) and goat anti‑human IgG (Biosource, AHI0501)

- Substrate: TMB (3,3′,5,5′-tetramethylbenzidine, Sigma)

- Stop solution: 1 M H₂SO₄

**Procedure:**

1. Coat each well with 100 µL of IFN-γ solution (10 µg/mL in PBS) and incubate overnight at 4°C.
2. Wash plates three times with PBS-Tween (0.05%).
3. Add 300 µL blocking buffer per well and incubate for 1 h at room temperature.
4. Wash three times. Add 100 µL of patient serum diluted 1:1000 in dilution buffer and incubate for 1 h at 37°C.
5. Wash three times. Add 100 µL of HRP-conjugated detection antibody (diluted as per manufacturer’s instructions) and incubate for 1 h at 37°C.
6. Wash three times. Add 100 µL TMB substrate and incubate for 15 min in the dark.
7. Stop reaction with 50 µL 1 M H₂SO₄and read absorbance at 450 nm.

**2. Plasma‑spiking neutralization assay**

**Reagents:**

- Human IFN-γ (R&D Systems)

- BD OptEIA Human IFN-γ ELISA Kit (BD Biosciences, 555142)

- Dilution series of patient serum (1:100 to 1:40,000)

**Procedure:**

1. Mix 100 µL of each serum dilution with 100 µL of IFN-γ solution (1000 pg/mL) and incubate for 1 h at 37°C.

2. Measure free IFN-γ concentration in the mixture using the BD OptEIA ELISA kit according to the manufacturer’s protocol.

3. The neutralizing titer is defined as the highest serum dilution that reduces free IFN-γ by 50% compared to the control well (IFN-γ without serum).

**3. Controls**

- Positive controls: sera from three previously confirmed cases of high-titer anti-IFN-γ autoantibody syndrome.

- Negative controls: pooled sera from 1000 healthy individuals undergoing routine health screening.

- Disease controls: sera from four patients with culture-proven NTM infection but no evidence of acquired immunodeficiency.

All assays were performed in duplicate. Inter‑assay variation was <10%.
